# Staging more important than grading? Evaluation of malignancy grading, depth of invasion, and resection margins in oral squamous cell carcinoma

**DOI:** 10.1007/s00784-020-03421-2

**Published:** 2020-06-29

**Authors:** Michael Wunschel, Miriam Neumeier, Kirsten Utpatel, Torsten E. Reichert, Tobias Ettl, Gerrit Spanier

**Affiliations:** 1 Department of Cranio-Maxillofacial Surgery, University Hospital Regensburg, Regensburg, Germany; 2 Institute of Pathology, University Regensburg, Regensburg, Germany

**Keywords:** Oral squamous cell carcinoma, Depth of invasion, Resection margin, Malignancy grading

## Abstract

**Objectives:**

The present study evaluated the predictive value of staging and grading parameters concerning the presence of lymph-node metastases, overall survival (OS), and relapse-free survival (RFS) of patients with oral squamous cell carcinoma (OSCC).

**Materials and methods:**

HE-stains of 135 surgically treated (R0) primary OSCCs were analyzed using a both microscopic and software-based approach. Depth of invasion (DOI) and resection margins (RM) were measured, and each case was graded according to the malignancy grading system as described by Anneroth et al. and Bryne et al. on two different sites of the tumor (surface and invasion front; TS and IF).

**Results:**

Parameters that could be identified as significant predictors of OS and RFS were UICC cancer stage (*p* = 0.009 and *p* = 0.012); pT-stage as defined in the 7th edition (*p* = 0.029 and 0.015) and, after restaging using DOI, 8th edition (*p* = 0.023 and *p* = 0.005) of the TNM classification of malignant tumors; the presence of lymphonodular metastases (LM) (*p* = 0.004 and *p* = 0.011); degree of keratinization (*p* = 0.029 and *p* = 0.042); and pattern of growth (*p* = 0.029 and *p* = 0.024) at the TS after applying a binary scale for both parameters. Also, when directly comparing the most extreme subgroups (scores 1 and 4) of lymphoplasmacytic infiltration at the IF, there was a significant difference in OS (*p* = 0.046) and RFS (*p* = 0.005). Invasion of blood vessels (*p* = 0.013) and perineural invasion (*p* = 0.023) were significantly associated with a lower OS. Age lower than 60 years (univariate *p* = 0.029, multivariate *p* = 0.031), infiltration of lymphatic vessels (*p* = 0.003), infiltration of nerves (*p* = 0.010), pT-stage (8th edition) (*p* = 0.014), degree of keratinization at the IF (*p* = 0.033), and nuclear polymorphism at the IF (*p* = 0.043) after conversion to a binary scale were found to be significant prognostic parameters regarding the presence of LM. DOI evolved as a significant predictor for OS (*p* = 0.006), RFS (*p* = 0.003), and LM (*p* = 0.032) in metric and grouped analysis.

**Conclusions:**

The current evaluation revealed depth of invasion as strongest histologic predictor of metastatic tumor growth, overall survival, and relapse-free survival in OSCC, confirming the current adaption of the T-classification. Other distinct histologic grading parameters investigated during this study can give valuable indications of a tumor’s potential aggressiveness, but the exact site, mode, and procedure need further exploration.

**Clinical relevance:**

Integrating measurement of DOI also into the pretherapeutic staging process could aid in treatment planning.

## Objectives

More than 500.000 new cases of head and neck squamous cell carcinoma (HNSCC) are diagnosed every year with a constant increase of oral squamous cell carcinoma (OSCC) and a decline in laryngeal and hypopharyngeal cancer over the past decade [1]. The annual incidence of oral cancer is estimated to be more than 300.000 and the annual mortality 145.000 deaths worldwide. OSCC constitutes 90 % of these cases [2]. Despite significant improvement in diagnostics and treatment since the 1970s [3], overall survival remains not satisfactory.

Current treatment options include surgical resection, radiation therapy, chemotherapy, and immunotherapy [4] based on national and international guidelines [5, 6]. Primary tumor resection with immediate reconstruction is usually performed in cases where localized disease is approachable with curative intention [6]. Adjuvant radiotherapy or systemic therapy with concurrent radiotherapy is used in cases with late-stage cancer or with adverse features like positive lymph nodes, extranodal extension, and involved or positive resection margins [7]. Patients with non-resectable tumors or medical conditions impeding surgery can be curatively treated with systemic therapy or irradiation or combination of both [7]. First-line therapy of very advanced, recurrent, or metastatic HNSCC currently consists of a combination of a platinum-based agent, fluorouracil and cetuximab [8]. Recently, new therapeutic options, which are directed at countering a tumor’s immune-evasion capabilities, for example, its expression of programmed death ligand 1 (PD-L1), are emerging [9]. Until now, monoclonal anti-PD-1 antibodies like nivolumab or pembrolizumab are recommended as second-line therapy in cases of tumor progression during or after first-line treatment [7], but more and more reports suggest superiority of these immune-based approaches to the established platinum-based treatment protocols [10, 11].

Among other criteria the choice of treatment vastly depends on patient’s tumor stage and grade. While histopathological grading according to the WHO classification of head and neck tumors often lacks significant prognostic value [12–14], clinical staging according to AJCC or UICC TNM classifications constitutes a major prognostic factor [12, 15, 16].

Several attempts have been made to improve the predictive accuracy introducing additional histologic parameters like tumor thickness, growth pattern, invasive front malignancy grading, tumor budding, or depth of invasion (DOI) [12, 17–20]. The latter has been included into the current (UICC, AJCC, eighth edition) TNM classification of malignant tumors of the oral cavity [15, 16].

The aim of the present study was to evaluate the predictive value of additional histologic parameters concerning the presence of lymph-node metastases (LM), overall survival (OS), and relapse-free survival (RFS) of patients with OSCC.

## Materials and methods

### Patients

The study comprises 135 adult patients diagnosed and treated for newly diagnosed OSCC at the Department of Cranio-Maxillofacial Surgery, University Hospital Regensburg between January 2013 and December 2016. Patients with previous neck dissection or primary systemic or radiotherapy of head and neck squamous cell carcinoma were excluded. All participants underwent surgical resection of the primary lesion to negative margins as well as neck dissection based on the clinical and radiologic findings. Only cases with definite histologically clear margins (R0) after surgery were included. Patients were staged according to the UICC guidelines of the 7th edition [21]. Patient data (age, sex, history of smoking and alcohol, tumor site, TNM stage, UICC stage, surgical therapy) were retrieved from the medical records for retrospective analysis.

Adjuvant treatment (radiotherapy and/or systemic therapy) was based on the recommendation of the multidisciplinary tumor board. Disease relapse was defined as local disease recurrence or distant metastasis by radiologic evidence with clinical correlation or histologic confirmation by biopsy. Follow-up data concerning recurrence-free survival (RFS) and overall survival (OS) were obtained from medical records, death certificates, registration offices, and the Clinical Cancer Registry of the Tumor Centre-Institute for Quality Management and Health Services Research, University of Regensburg, Germany.

### Specimen and histological evaluation

Standard formalin-fixed, paraffin-embedded tissue samples of all patients were retrieved from the archive of the Institute of Pathology, University of Regensburg, Germany. For HE-staining, 4 μm sections were deparaffinized with xylene and ethanol and stained with HE according to standard protocol. For analysis an Axiostar plus microscope (Carl Zeiss, Göttingen, Germany) with a Cl 10× ocular (Carl Zeiss, Göttingen, Germany) and CP-Achromat microscope 5×, 10×, and 40× objectives (Carl Zeiss, Göttingen, Germany) was used. All available HE-slides with tissue of the primary tumor were analyzed, and the one most suitable slide for each site investigated (tumor surface (TS) and invasion front (IF)) was selected. Selection criteria included quantity of visible tumor cells, quality of stain, and lack of preparation artifacts or other structural damages to the tissue. Optimal slides were scanned in a Pannoramic 250 FLASH III (Sysmex, Norderstedt, Germany) scanner, and accordant JPEG-format files (2520 × 1481 pixels × 24 Bit) were generated. Digital images were evaluated by using Caseviewer Software (Sysmex, Norderstedt, Germany).

**Fig. 1:**
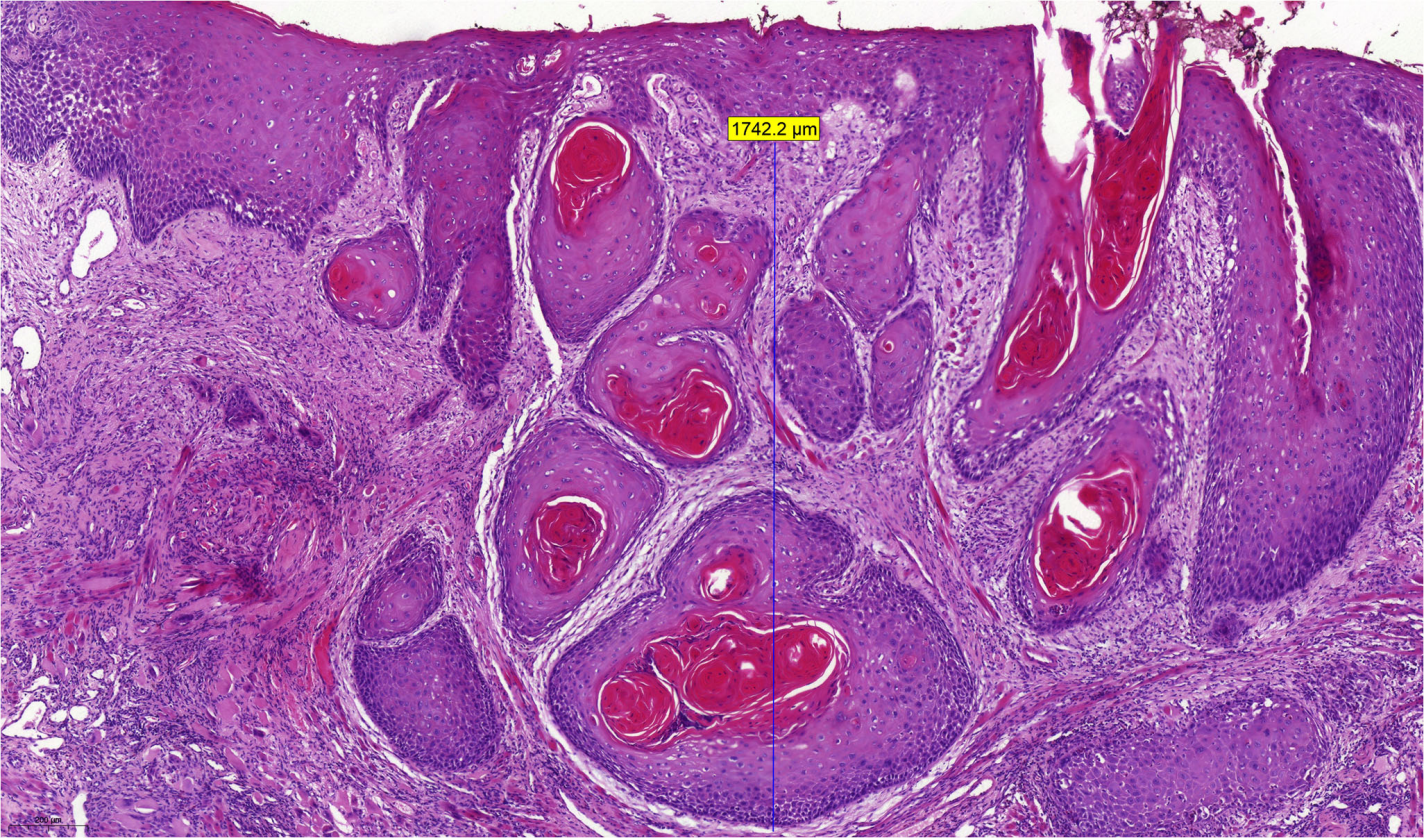
Histopathological slide of an oral squamous cell carcinoma showing measurement of DOI (magnification × 7.0)

**Fig. 2:**
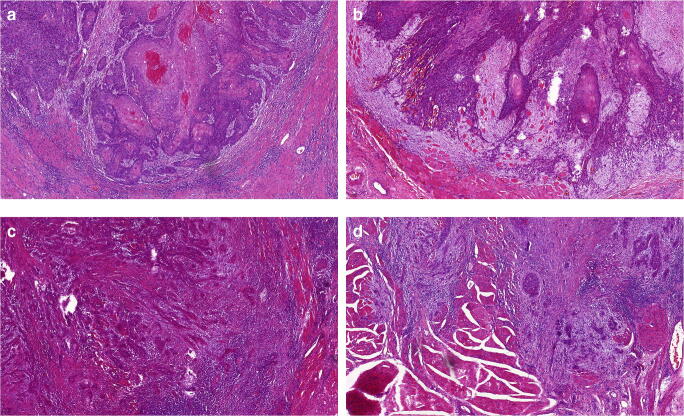
Histopathological slides of oral squamous cell carcinoma showing examples for different patterns of invasion (magnification × 8.0): **A** grade 1, pushing, well-delineated infiltrating borders; **B** grade 2, infiltrating, solid cords, bands, and/or strands; **C** grade 3, small groups or cords of infiltrating cells (*n* > 15); and **D** grade 4, marked and widespread cellular dissociation in small groups and/or in single cells (*n* < 15)

Depth of invasion (DOI) and resection margins (RM) were measured with micrometer precision. DOI was defined as the maximum distance from the basal membrane to the tumor’s deepest margin [16] (see Fig. 1). DOI was then used to restage tumors according to the 8th edition of the TNM classification of malignant tumors [15] creating a “new pT-stage”-variable. RM was defined as the minimum distance of a tumor margin to the border of a tissue specimen. Oriented tumor-free safety margin excisions were added to the corresponding value. Each case was then graded according to the malignancy grading system as described by Anneroth et al. [20] and Bryne et al. [18, 22]. Each parameter including degree of keratinization, nuclear polymorphism, number of mitoses per high power field (HPF), pattern of growth/invasion, and lymphoplasmacytic invasion was given a grade/score of 1–4 [23] (Table 1). Further analysis was carried out by dichotomizing the results of each parameter by combining scores/grades 1 and 2 into a low-risk group 1 and scores/grades 3 and 4 into a high-risk group 2. In contrast to the concept of “invasive cell grading (ICG)” [23], we investigated tumor surface (TS)—as obtained from biopsies—and invasion front (IF) separately, if sufficient material for distinct observation was available. Cells at the tumor surface were also grouped according to the criteria previously described as “pattern of invasion” [20], referring to this investigation as “pattern of growth.” In cases, where distinct analysis was not possible, the data were attributed to the invasion front subgroup. See Fig. 2 with examples of the four categories of invasion patterns. Shown are different grades of invasion patterns representing differences in cell-to-cell cohesiveness from intact, well-delineated margins in differentiated tumors (grade 1) to single invasive cells in undifferentiated tumors (grade 4). The number of mitoses was counted reviewing ten HPF and recorded as exact number and coded as a score as shown in Table 1. All scores were then summoned up into one malignancy score for tumor surface and invasion front respectively [22].

**Table 1:** Histological malignancy grading system according to Anneroth (1987) [20] and Bryne (1992) [23]

Morphological feature	Score			
	1	2	3	4
Degree of keratinization	Highly keratinized(> 50% of the cells)	Moderately keratinized (20–50% of the cells)	Minimal keratinization(5–20% of the cells)	No keratinization(0–5%, of the cells)
Nuclear polymorphism	Little nuclear polymorphism(> 75% mature cells)	Moderately abundant nuclear polymorphism(50–75% mature cells)	Abundant nuclear polymorphism (25–50% mature cells)	Extreme nuclear polymorphism(0–25% mature cells)
Number of mitoses (high power field)	0–1	2–3	4–5	>5
Pattern of growth/invasion	Pushing, well-delineated infiltrating borders	Infiltrating, solidcords, bands, and/or strands	Small groups orcords of infiltrating cells (*n* > 15)	Marked and widespread cellular dissociation in small groups and/or in single cells (*n* < 15)
Lymphoplasmacytic infiltration	Marked	Moderate	Slight	None

Histologic investigation and grading were independently performed by three investigators (MW, MN, and KU) without knowing the clinical follow-up of the patients in a blinded approach. In case of divergent assessment, consent was obtained after joint re-evaluation.

### Statistical analysis

Metric variables were analyzed for differences in their means, using Student’s *t* test in case of log-normal distribution, otherwise using Mann–Whitney *U* test. Independence or correlation of categorical variables was analyzed using Pearson’s chi-squared test and reporting the Phi-coefficient. Multivariate correlation analysis was performed using a binary logistic regression model. OS and RFS time were calculated from date of resection to date of death, date of recurrence, or date last alive until cut-off date June 30, 2019. Survival analyses were performed using univariate Kaplan–Meier and multivariate Cox regression method. Differences in outcome estimates were tested using the log-rank test. Results were reported with hazard ratios (HRs) and 95% confidence interval (CI). A *p* < 0.05 was considered significant for all tests. All data were anonymized, and analyses were performed using IBM SPSS Statistics Version 25.0 (IBM Corp., Armonk, N.Y., USA).

## Results

After applying the study’s criteria, 135 patients, thereof 94 males and 41 females, were included. Mean age at the time of the operation was 62.69 years. The majority of patients reported a history of smoking or alcohol, or a combination of both and OSCC in this collective was most frequently located in the tongue or floor of mouth. Additional patient and tumor data are shown in Tables 2 and 3.

**Table 2:** Patient and tumor data

Parameter	Variable	Value	Test result (overall survival)	Test result (relapse-free survival)
Observation period (days)				
	Mean (range)	1193.83 (17–2228)		
Gender			*χ*^2^(1) = 0.023, *p* = 0.880	*χ*^2^(1) = 0.019, *p* = 0.890
	Male	94 (69.6%)		
	Female	41 (30.4%)		
Age (years)			Exp(*B*) = 1.004, *p* = 0.751	Exp(*B*) = 1.008, *p* = 0.480
	Mean (range)	62.69 (27–90)		
History of smoking			*χ*^2^(1) = 1.273, *p* = 0.259	*χ*^2^(1) = 0.675, *p* = 0.411
	Yes	89 (65.9%)		
	No	46 (34.1%)		
History of alcohol			*χ*^2^(1) = 0.337, *p* = 0.562	*χ*^2^(1) = 0.932, *p* = 0.334
	Yes	80 (59.3%)		
	No	55 (40.7%)		
Localization of tumor			*χ*^2^(5) = 3.939, *p* = 0.558	*χ*^2^(5) = 4.566, *p* = 0.471
	Buccal plane	15 (11.1%)		
	Maxilla	6 (4.4%)		
	Mandible	20 (14.8%)		
	Palate	6 (4.4%)		
	Tongue	43 (31.9%)		
	Floor of mouth	45 (33.3%)		
Depth of invasion (mm)			*Exp(B) = 1.067, p = 0.015*	*Exp(B) = 1.082, p = 0.001*
	Mean (range)	8.505 (0.997–25.867)		
Safety margin (mm)			Exp(*B*) = 0.924, *p* = 0.117	Exp(*B*) = 0.950, *p* = 0.244
	Mean (range)	6.024 (0.172–16.995)		

Significant results are shown in italics

**Table 3:** Univariate analysis of overall (OS) and recurrence-free (RFS) survival. Only statistically significant parameters are shown

Parameter		Number	OS			RFS		
			2YSR (%)	5YSR (%)	Test result	2YSR (%)	5YSR (%)	Test result
UICC					*χ*^*2*^*(3) = 11.672, p = 0.009*			*χ*^*2*^*(3) = 10.979, p = 0.012*
	I	38 (28.1%)	97.4 ± 2.6	74.1 ± 9.1		86.8 ± 5.5	65.7 ± 9.7	
	II	20 (14.8%)	90.0 ± 6.7	63.0 ± 16.0		85.0 ± 8.0	53.0 ± 15.0	
	III	22 (16.3%)	59.1 ± 10.5	47.3 ± 11.2		54.5 ± 10.6	48.5 ± 11.0	
	IV	55 (40.7%)	63.6 ± 6.5	54.3 ± 7.0		52.7 ± 6.7	44.5 ± 6.8	
pT (7th Ed.)					*χ*^*2*^*(3) = 9.011, p = 0.029*			*χ*^*2*^*(3) = 10.418, p = 0.015*
	1	51 (37.8%)	90.2 ± 4.2	67.9 ± 7.9		78.4 ± 5.8	63.0 ± 8.1	
	2	39 (28.9%)	76.9 ± 6.7	57.0 ± 11.2		69.2 ± 7.4	45.4 ± 11.1	
	3	13 (9.6%)	38.5 ± 13.5	38.5 ± 13.5		30.8 ± 12.8	30.8 ± 12.8	
	4a	32 (23.7%)	68.8 ± 8.2	57.8 ± 9.0		62.5 ± 8.6	49.0 ± 9.0	
pT (8th Ed.)					*χ*^*2*^*(3) = 9.576, p = 0.023*			*χ*^*2*^*(3) = 12.997, p = 0.005*
	1	28 (20.7%)	92.9 ± 4.9	74.5 ± 9.4		85.7 ± 6.6	67.5 ± 9.9	
	2	34 (25.2%)	85.3 ± 6.1	66.5 ± 10.5		73.5 ± 7.6	59.9 ± 11.5	
	3	32 (23.7%)	56.3 ± 8.8	42.4 ± 9.7		43.8 ± 8.8	31.2 ± 8.8	
	4a	32 (23.7%)	68.8 ± 8.2	57.8 ± 9.0		62.5 ± 8.6	49.0 ± 9.0	
V					*χ*^*2*^*(1) = 6.128, p = 0.013*			*χ*^2^(1) = 2.439, *p* = 0.118
	0	131 (97.0%)	77.9 ± 3.6	61.5 ± 4.9		68.7 ± 4.1	53.6 ± 4.9	
	1	4 (3.0%)	25.0 ± 21.7	25.0 ± 21.7		25.0 ± 21.7	25.0 ± 21.7	
Pn					*χ*^*2*^*(1) = 5.147, p = 0.023*			*χ*^2^(1) = 5.147, *p* = 0.120
	0	120 (88.9%)	80.0 ± 3.7	63.1 ± 5.2		70.0 ± 4.2	54.5 ± 5.2	
	1	15 (11.1%)	46.7 ± 12.9	40.0 ± 12.6		46.7 ± 12.9	40.0 ± 12.6	
Lymphonodular metastasis					*χ*^*2*^*(1) = 8.472, p = 0.004*			*χ*^*2*^*(1) = 6.434, p = 0.011*
	neg.	85 (63.0%)	85.9 ± 3.8	66.7 ± 6.3		77.6 ± 4.5	57.1 ± 6.4	
	pos.	49 (36.3%)	61.2 ± 7.0	50.4 ± 7.6		51.0 ± 7.1	46.1 ± 7.3	
Depth of invasion					*χ*^*2*^*(2) = 7.701, p = 0.021*			*χ*^*2*^*(2) = 10.614, p = 0.005*
	< 5 mm	37 (29.6%)	94.6 ± 3.7%	75.5 ± 7.8%		89.2 ± 5.1%	70.4 ± 8.1%	
	5 ≤ DOI < 10 mm	43 (34.4%)	76.7 ± 6.4%	62.0 ± 9.0%		65.1 ± 7.3%	52.9 ± 9.2%	
	≥ 10 mm	45 (36.0%)	57.8 ± 7.4%	48.8 ± 7.8%		46.7 ± 7.4%	38.5 ± 7.5%	
Tumor surface								
Degree of keratinization (dichotomized)					*χ*^*2*^*(1) = 4.752, p = 0.029*			*χ*^*2*^*(1) = 4.144, p = 0.042*
	1	89 (85.6%)	78.7 ± 4.3	65.7 ± 5.8		68.5 ± 4.9	55.7 ± 5.9	
	2	15 (14.4%)	53.3 ± 12.9	38.9 ± 12.9		40.0 ± 12.6	33.3 ± 12.2	
Pattern of growth (dichotomized)					*χ*^*2*^*(1) = 4.753, p = 0.029*			*χ*^*2*^*(1) = 4.582, p = 0.032*
	1	37 (35.6%)	91.9 ± 4.5	74.9 ± 8.0		81.1 ± 6.4	64.8 ± 8.5	
	2	67 (64.4%)	65.7 ± 5.8	55.9 ± 6.4		55.2 ± 6.1	46.9 ± 6.5	

Significant results are shown in italics

### Univariate survival analysis of standard parameters

Mean observation period was 1193.83 days. Estimated mean overall survival was 1618.83 (± 70.28; 1481.08–1756.58 95% CI) days, resulting in a 2-year survival rate (2YSR) of 76.3% (± 3.7%) and a 5-year survival rate (5YSR) of 60.4% (± 4.9%). Estimated mean RFS was 1449.86 (± 77.42; 1298.11–1601.61 95% CI) days. Mean time to relapse was 360 (± 283; 254–465 95% CI; 39 min, 1376 max) days.

See Tables 2 and 3 for results of univariate survival analysis.

For statistical analysis, cancer stages IVa and IVc as defined in the 7th edition of UICC cancer manuals [21] were combined to one stage IV, because there was only one case with stage IVc. The same applied for G4 grade, which—in concordance with international standards [16]—was counted in the G3-subgroup.

Following standard staging parameters showed significant association with OS and RFS: UICC cancer stage, pathohistological pT-stage, as defined in the 7th edition of the TNM classification of malignant tumors [21] and the presence of lymphonodular metastases (LM). The number of infiltrated lymph nodes was a significant predictor of OS (Exp(*B*) = 1.201, *p* < 0.001) and RFS (Exp(*B*) = 1.149, *p* < 0.001).

Invasion of blood vessels and perineural invasion were associated with significantly worse overall survival. This association was not observed for RFS.

All other standard and staging parameters did not show statistical significance for OS or RFS.

### Univariate survival analysis of additional histological parameters

#### Depth of invasion and resection margin

**Fig. 3:**
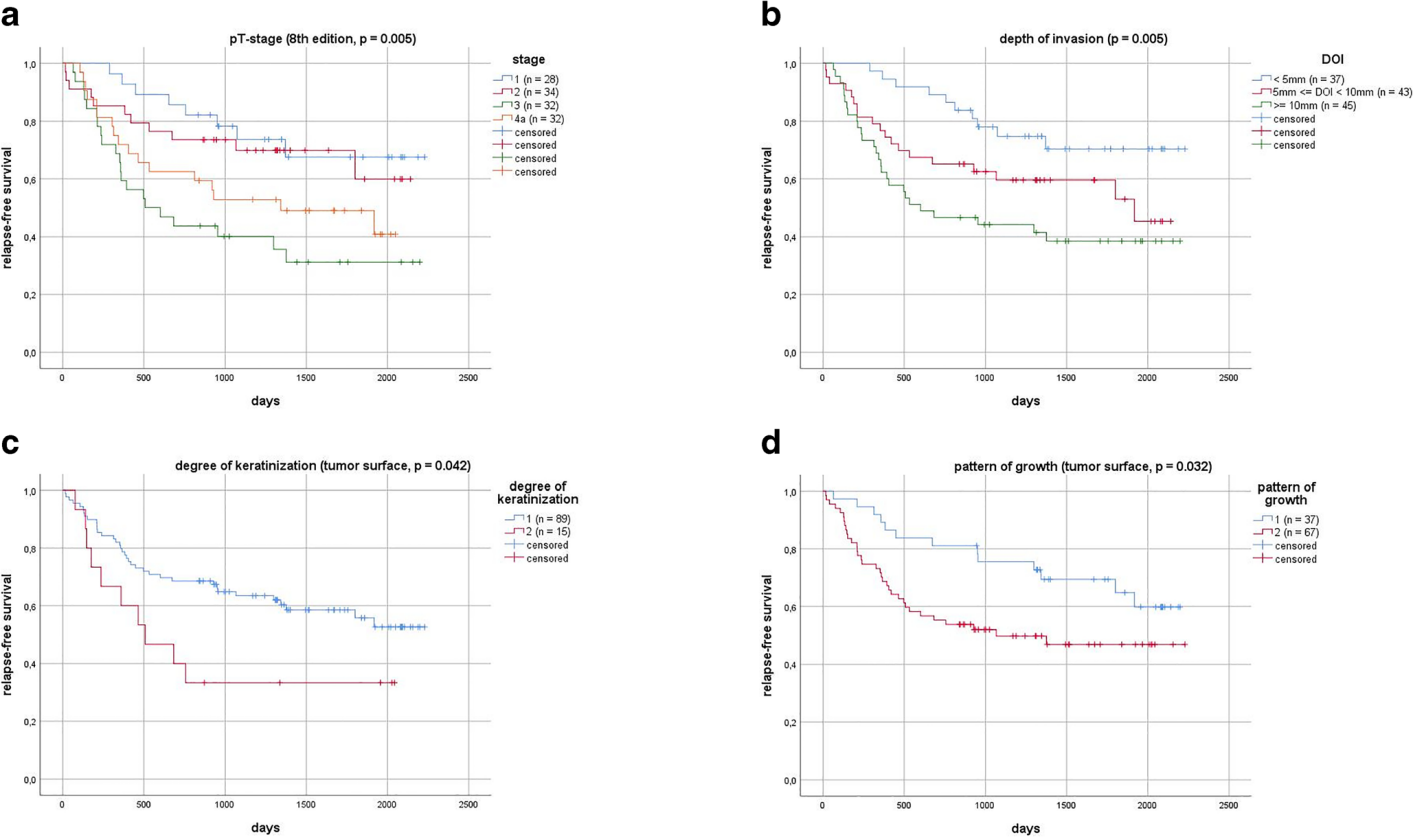
Kaplan–Meier overall survival for pT-stage (**A**, *p* = 0.023), depth of invasion (**B**, *p* = 0.021), degree of keratinization (**C**, *p* = 0.029), and pattern of growth (**D**, *p* = 0.029)

**Fig. 4:**
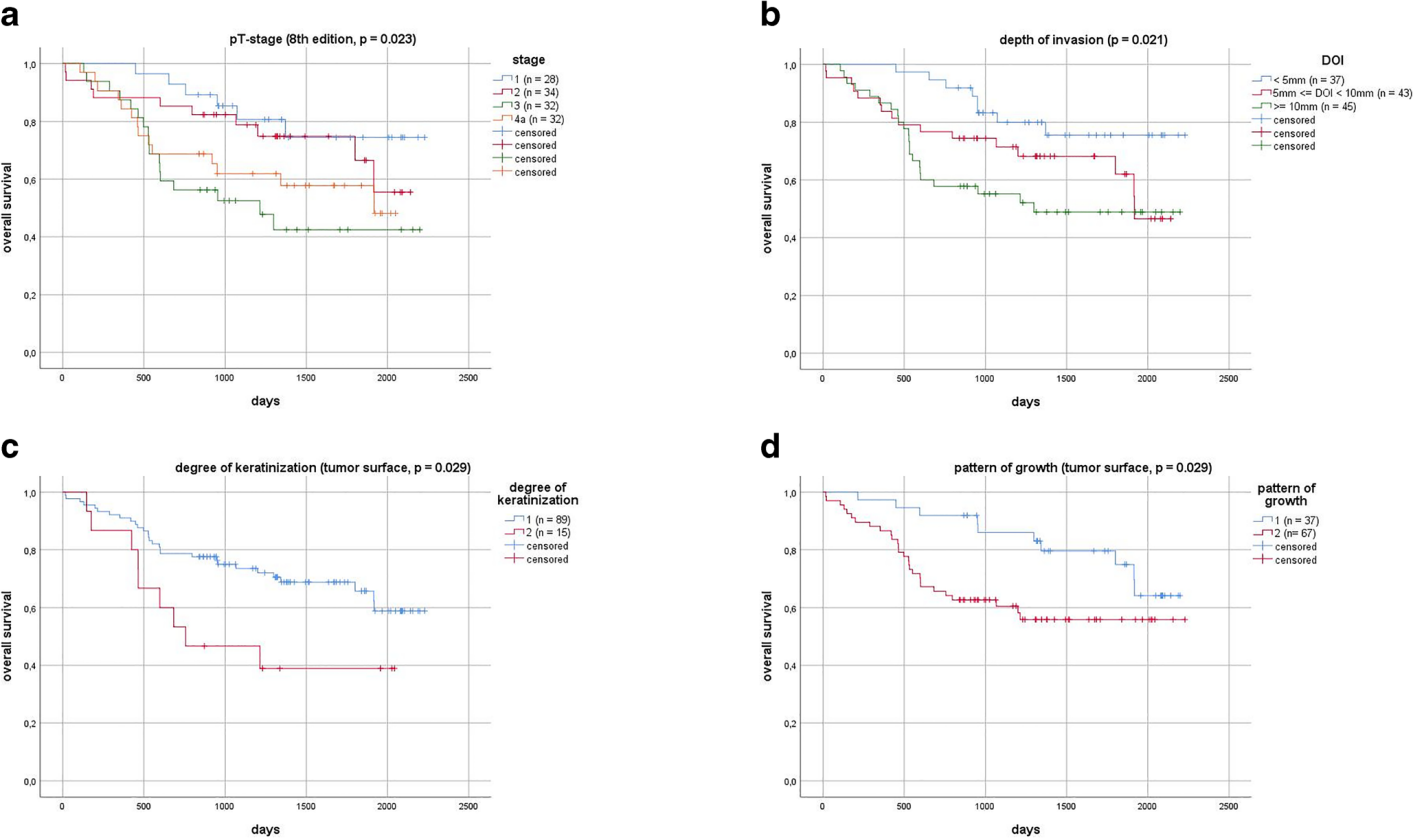
Kaplan–Meier relapse-free survival for pT-stage (**A**, *p* = 0.005), depth of invasion (**B**, *p* = 0.005), degree of keratinization (**C**, *p* = 0.042), and pattern of growth (**D**, *p* = 0.032)

In Cox regression analysis, DOI was identified as a significant parameter of OS and RFS. A cut-off value separating low-risk and high-risk groups was calculated. In this collective, a DOI cut-off of 6 mm was ideal to separate these two groups (OS: *χ*^2^(1) = 7.601, *p* = 0.006; RFS: *χ*^2^(1) = 8.912, *p* = 0.003). In literature, most commonly a cut-off of 5 mm is described [24, 25]. When applying this value in order to separate the groups, DOI was still a significant parameter estimating OS (*χ*^2^(1) = 5.980, *p* = 0.014) and RFS (*χ*^2^(1) = 7.860, *p* = 0.005). Additionally, all cases were then divided up into subgroups of ≤ 5 mm, 5 < DOI ≤ 10 mm and > 10 mm according to the current TNM classification [16, 21] (see Figs. 3 and 4). This categorical division of DOI was also a significant predictor of OS and RFS.

Resection margin (RM) on the other hand could not be identified as a significant predictor of OS or RFS. Patients were then summarized into subgroups (RM ≤ 1 mm, 1 mm < RM ≤ 5 mm, > 5 mm) according to values previously published as significant prognostic indicators [26, 27], also yielding no significant result.

When restaging tumors according to the 8th edition of TNM classification of malignant tumors [15], using DOI as an additional parameter, new pT-stage was a significant predictor of OS and RFS, pointing towards a slight improvement in predictive power, when compared with the old classification (Figs. 3 and 4). Restaging led to an upstaging of pT1 to pT2 in 25.5% of the cases, pT1 to pT3 in 2.9%, and pT2 to pT3 in 30.8%. This new pT-stage and the information of extracapsular spread (ECS) obtained from the histopathological reports were then used to evaluate UICC (8th edition) [15] cancer stage for every case. There was an upstaging from stage I to stage II in 6.7% of the cases, from stage II to stage III in 4.4%, from stage III to stage IVa in 0.7%, from stage III to stage IVb also in 0.7%, and from stage IVa to stage IVb in 10.4%.

#### Malignancy grading of tumor surface

Degree of keratinization on a 4-point scale could not be identified as a significant predictor of OS or RFS. A binary scale was then applied by grouping together patients with highly (score = 1) or moderately (score = 2) keratinized TS in one new subgroup (group 1) and patients with minimal (score = 3) or no (score = 4) keratinization at the TS into another subgroup (group 2). Difference in OS in both groups with a 2YSR in group 1 of 78.7 ± 4.3% and in group 2 of 53.3 ± 12.9% and a 5YSR in group 1 of 65.7 ± 5.8% and in group 2 of 38.9 ± 12.9% was significant (*χ*^2^(1) = 4.752, *p* = 0.029) (Fig. 3). RFS did also differ significantly after dichotomizing the results (*χ*^2^(1) = 4.144, *p* = 0.042) (Fig. 4).

Pattern of growth on a four-point scale could not be identified as a significant predictor of OS or RFS. After dichotomizing this variable, there was a significant difference in OS (*χ*^2^(1) = 4.753, *p* = 0.029) (Fig. 3) and RFS (*χ*^2^(1) = 4.582, *p* = 0.032) (Fig. 4) between the new subgroups. 2YSR (OS) in new group 1 was 91.9 ± 4.5% and 65.7 ± 5.8% in group 2; 5YSR were 74.9 ± 8.0% and 55.9 ± 6.4%, respectively. Analyzing the single subgroups, there was a significant difference in OS of patients with grade 1 (pushing, well-delineated borders) and grade 2 (infiltrating, solid cords, bands and/or strands) tumors when compared to grade 4 tumors (cellular dissociation) (*χ*^2^(1) = 4.436, *p* = 0.035, and *χ*^2^(1) = 5.060, *p* = 0.024, respectively).

There was no difference in OS or RFS found when comparing patients grouped according to nuclear polymorphism, number of mitoses, or lymphoplasmacytic infiltration at the TS.

All measurements performed on the TS were then added up to create a combined malignancy score with a theoretical range of 5–20. Univariate analysis of this combined tumor surface malignancy score revealed it to be a significant predictor of OS (Exp(*B*) = 1.160, *p* = 0.019) and RFS (Exp(*B*) = 1.160, *p* = 0.019). A cut-off value of 13 was ideal to separate low-risk and high-risk groups concerning OS (*χ*^2^(1) = 5.295, *p* = 0.021) and RFS (*χ*^2^(1) = 15.886, *p* < 0.000).

#### Malignancy grading of invasion front

There was no significant difference in OS or RFS when analyzing lymphoplasmacytic infiltration at the site of the IF. However, when looking at the most extreme subgroups with either a marked lymphoplasmacytic infiltration (score = 1) or no lymphoplasmacytic infiltration at all (score = 4), there was a significant difference in OS (*χ*^2^(1) = 3.973, *p* = 0.046). Furthermore, a marked (score = 1) and moderate (score = 2) lymphoplasmacytic infiltration was associated with a significantly higher RFS than in cases, where no lymphoplasmacytic infiltration (score = 4) was present (*χ*^2^(1) = 8.052, *p* = 0.005, and *χ*^2^(1) = 4.192, *p* = 0.041, respectively).

There was no significant difference in OS or RFS, when comparing patients grouped according to degree of keratinization, nuclear polymorphism, number of mitoses, or pattern of invasion at the IF.

Also, the combined IF malignancy grading score was not a significant predictor of OS (Exp(*B*) = 1.097, *p* = 0.131) or RFS (Exp(*B*) = 1.093, *p* = 0.112).

### Multivariate survival analysis

All single parameters (new pT1 + 2 vs 3 + 4, V, Pn, LM, degree of keratinization 1 + 2 vs 3 + 4, pattern of growth 1 + 2 vs 3 + 4, DOI with a cut-off of 5 mm) that had shown significant impact on OS were then integrated into a multivariate Cox regression model. In this analysis, only V1 was identified as independent, significant factor associated with reduced OS (Table 4). When performing this analysis regarding RFS, no parameter could be identified as a significant factor.

**Table 4:** Multivariate analysis (overall survival)

Parameter	Number	Exp(B)	Test result
Depth of invasion ≥ 5 mm	81	2.815	*p* = 0.108
Degree of keratinization 3 + 4 (surface)	15	1.799	*p* = 0.145
Pattern of growth 3 + 4 (surface)	66	1.827	*p* = 0.147
pT3 + 4	56	0.919	*p* = 0.838
Lymponodular metastasis pos.	40	1.684	*p* = 0.136
V1	3	5.136	*p = 0.014*
Pn1	14	1.962	*p* = 0.105

Significant results are shown in italics

#### Predictive value of standard and additional histologic parameters concerning the presence of lymphonodular metastases

Of all standard and staging parameters, younger age (*T* = 2.220, *p* = 0.029), infiltration of lymphatic vessels (*χ*^2^(1) = 8.788, *p* = 0.003, *ϕ* = 0.256), and infiltration of nerves (*χ*^2^(1) = 6.597, *p* = 0.010, *ϕ* = 0.222) were significantly correlated with LM. The ideal cut-off to discriminate low- and high-risk groups for LM regarding age was 60 years (*χ*^2^(1) = 6.610, *p* = 0.010, *ϕ* = 0.222). Rate of LM was 50.0% in patients younger than 60 years and 28.0% in patients with an age of 60 years or older.

Mean DOI in patients without LM was 7.71 mm compared to 9.73 mm in patients with the presence of LM. This difference was significant (*T* = − 2.163, df = 122, *p* = 0.032). A cut-off value off 5 mm was found to be ideal to separate low-risk and high-risk groups (*χ*^2^(1) = 4.600, *p* = 0.032, *ϕ* = 0.193). Rate of LM was 24.3% in the group with DOI < 5 mm and 44.8% in the group with DOI ≥ 5 mm.

New pT-stage according to the 8th edition of UICC cancer staging manual was a significant parameter associated with LM (*χ*^2^(3) = 10.634, *p* = 0.014, *ϕ* = 0.292).

Degree of keratinization at the IF on a 4-point and a 2-point scale was a significant predictor regarding the presence of lymphonodular metastatic spread (*χ*^2^(3) = 8.715, *p* = 0.033, *ϕ* = 0.267, and *χ*^2^(1) = 4.802, *p* = 0.028, *ϕ* = 0.198). There were significantly more patients without LM in score-group 1 (23.9%) and more patients with LM in score-group 4 (66.7%).

The parameter nuclear polymorphism at the IF, after regrouping all cases according to a binary scale, was significantly associated with LM (*χ*^2^(1) = 4.106, *p* = 0.043, *ϕ* = 0.183). Rate of LM in group 1 was 30.7% and 48.9% in group 2.

All other investigated parameters were not significantly correlated with the presence of LM.

All categorial parameters that had been found to be significantly correlated with the presence of LM were then added to multivariate regression model (see Table 5). Here, the age group below 60 years and new pT-stage 3 could be identified as independent, significant predictors of LM.

**Table 5:** Association with lymphonodular metastasis (multivariate)

Parameter	Number	Exp(B)	test result
Age < 60 years	46	2.569	*p = 0.031*
pT1 (indicator)	28		*p* = 0.087
pT2	31	2.308	*p* = 0.194
pT3	30	5.289	*p = 0.012*
pT4	31	1.994	*p* = 0.282
Pn1	14	0.987	*p* = 0.986
L1	10	4.125	*p* = 0.121
Degree of keratinization 3 + 4 (invasive front)	41	1.403	*p* = 0.487
Nuclear polymorphism 3 + 4(invasive front)	47	1.816	*p* = 0.201

Significant results are shown in italics

## Discussion

The interpretation of our results regarding the malignancy grading system revealed a heterogenous picture.

### Number of mitoses

The number of mitoses at both sites (surface and invasion front) reviewed and analyzed as exact number and coded as a score was not a suitable value to predict or estimate any outcome parameter. This finding corresponds to previous observations [17, 23, 28], though a high mitotic activity is seen as a surrogate marker for poorly differentiated tumors associated with poor prognosis [29].

### Nuclear polymorphism

Quite the same applied for our results on nuclear polymorphism. We did not see any statistical association of this parameter with any of the evaluated survival parameters. However, after combining the two lower and higher grades, the high-grade (3 + 4) group of nuclear polymorphism at the site of the invasive front showed significantly more lymphonodular metastases compared to the low-grade (1 + 2) group. This corresponds well to the biological model of loss of differentiation and metastasizing and was reported before [30]. However, the discrepancy in the present study’s findings could partly be explained with nuclear polymorphism being the most unreliable or uncertain parameter, as explained in one interrater study, where it reached the poorest interobserver agreement [31].

### Lymphoplasmacytic infiltration

Lymphoplasmacytic infiltration is seen as inflammatory response of the host against tumor growth [18, 32], and recently, immune-modulation–based strategies have become of increasing interest in the therapy of OSCC [33–35]. In this collective, patients with no lymphoplasmacytic infiltrate present at the site of the invasive front had the lowest OS when compared to patients with a marked lymphoplasmacytic infiltrate and the lowest RFS when compared to patients with either a marked or moderate inflammatory infiltrate. These results could not be seen when grading the more superficial cell layers of the tumor, possibly giving another hint that lymphocytes invading the tumor mass do indeed represent the host’s counter-tumor activity [36–39].

### Degree of keratinization

Keratinization or the lack thereof is often regarded as representing the grade of tissue differentiation of a tumor [20, 40] and several studies described a high degree of keratinization being associated with a better prognosis [39, 41] and a tendency to lower keratinization in cases of tumor recurrence [39]. In the present study, patients with higher degrees of keratinization (scores 1 and 2 combined) at the tumor surface showed a higher OS and RFS than patients with only minimal or no keratinization (scores 3 and 4 combined). In contrast, when assessing keratin formation at the invasive front, there was no significant difference between the groups. Also, the presence of LM was significantly lower in patients with highly keratinized cell formations at the invasive front and significantly higher in patients with no keratin formation at this site. Similar findings have been reported by other groups [28, 31].

### Pattern of invasion

Grading systems for malignant diseases are supposed to describe relevant morphological characteristics of the tumor but should also be easy to use and reproducible [12, 31]. Concerning OSCC, classical categories to describe the whole tumor’s morphology had been “structure,” “pattern,” and “mode of invasion” and these categories were subsumed by Anneroth under a new category “mode of invasion” in order to easily describe tumor growth and tumor-host interaction [20]. In contrast to Bryne’s modification of the malignancy grading [22], all categories were applied to both the tumor surface and the invasive front. Also, in contrast to former reports [18, 22], pattern of invasion (POI) in this study was not significantly correlated to any prognostic values. Pattern of growth (POG) at the site of the tumor surface however did reveal a higher OS in score-group 1 and a higher OS and RFS in score-group 2, when compared with patients with score = 4. After modifying this variable and creating a binary scale, there was a significant difference in OS and RFS between the two groups.

### Staging parameters

In order to predict prognosis and choose the necessary treatment options, not only histological grading but also preoperative and postoperative tumor stage is very important and needs to be considered. All parameters, which are currently applied during routine work-up as staging variables and are ultimately used to define possible postoperative treatment, were evaluated. Infiltration of blood vessels and perineural Infiltration were found to be significant predictors of OS, while UICC stage and T-stage according to the 7th edition of the TNM classification of malignant tumors [21] were found to be significant predictors of OS and RFS. Infiltration of lymphatic vessels, perineural invasion, and an age younger than 60 years were significantly associated with LM, the latter in univariate and multivariate analysis. The survival rates presented in the present study roughly correspond to those reported in literature [42–44]. However, pT3 stage in this collective was associated with the lowest OS and RFS with a lower 2YSR and 5YSR than pT4a stage. Like other authors, we interpret this fact as small tumors showing bone invasion and therefore formally qualifying for pT4a stage [45]. In contrast to pT3-tumors deeply infiltrating the soft tissues, these cases can be very accessible for sufficient surgical treatment with a safe and complete tumor resection. The importance of initial free resection margins has been reported elsewhere [46]. To increase selectivity and implement results of ongoing research, there have been some changes in the current, 8th edition of the respective TNM staging manuals, mainly with introduction of DOI as a stage defining parameter [15, 16, 45]. Using the values gathered during the reported investigations, all cases were restaged to get a “new pT-stage.” There are some indications that this reclassification did indeed slightly improve selectivity and thereby predictive power of the pT classification, and only the newly staged groups were significant predictors of LM. Still, the irregularities regarding OS and RFS in late-stage cancer (T3 and T4) mentioned earlier persist.

### Resection margins

To address these issues, also two staging parameters, DOI and RM, which can be assessed during pathohistological examination of HE-slides, were evaluated separately. In concordance with other publications [43, 47, 48] resection margin could not be identified as a significant predictor of survival or LM, even when summarizing patients into subgroups (RM ≤ 1 mm, ≤ 5 mm, > 5 mm) according to values previously published as significant prognostic indicators [26, 27]. These findings can possibly be explained with complete (R0) tumor resection being achieved in all patients of this collective and because mean RM was 6.0 mm and the number of patients with involved (RM ≤ 1 mm) margins was only 5. On the other hand, relapse does occur in patients with diagnosed free margins, so additional risk factors have to be evaluated [43, 49]. As mentioned before, positive frozen section margins or free margins only achieved by revision surgery are risk factors for local tumor relapse [46].

### Depth of invasion

DOI on the other hand was found to be an independent predictor of OS, RSF, and LM. These findings concur with results published elsewhere [24, 50]. Very often, cut-off-values defining the at-risk-group of 4 mm [49, 51–53] or 5 mm [24] are reported and have even been implemented into UICC and AJCC pT-stages [15, 16], but there are also hints of little difference between these two groups, lastly advising the use of 5 mm out of practical considerations [25]. In the present study, DOI as an absolute value was identified of being an independent risk factor with a cut-off value between low-risk and high-risk groups at 6 mm. Similarly, DOI has also been investigated in preoperative cancer biopsies with promising results [51]. It should however be emphasized that the tissue specimens are required to fulfill certain quality standards in order to be evaluated properly [49, 51, 54]. Additionally, definitions of DOI may vary [50, 55] and there is a widespread uncertainty in clearly separating DOI from measuring tumor thickness [25]. The concept of calculating a “relative DOI” by relating measured DOI with site-specific characteristics like the thickness of submucosal tissue has been promoted [49] but needs further investigation. Because of its relevance regarding outcome and the presence of LM, preoperative and adjuvant choice of therapeutic options, especially elective neck dissection, should take DOI into consideration. Though, due to contradicting reports [56, 57], it remains unclear, how to identify patients, especially with early-stage cancer, that would benefit from elective neck dissection. Several methods of evaluating DOI during the pretherapeutic staging process have been evaluated. There was a good correlation reported between clinical examination, MRI findings, and pathohistological results in tumors with DOI > 5 mm [58]. There are also data suggesting that intraoral ultrasound could be superior to other diagnostic entities and also applicable to early-stage cancer [59, 60].

## Conclusions

In conclusion, some specific parameters like the mitotic count alone seem not to be valid prognostic parameters, while the heterogenous picture revealed, when assessing lymphoplasmacytic infiltration or degree of keratinization, suggests that these features could be helpful in predicting tumor-specific prognosis. The exact mode and procedure though remain unclear and need further evaluation. Analyzing OSCC’s patterns of growth gives valuable diagnostic and prognostic information, but a valid scoring and interpretation system has yet to be created. Recently, similar or derived concepts like tumor budding and cell nests have been evaluated for other entities [61, 62] and OSCC [12, 17, 51, 63] with promising results. Dichotomizing complex histological grading systems partly sacrifices diagnostic accuracy, but it can increase reproducibility and specificity of the diagnosis [31, 64, 65]. We think further efforts should be made to evaluate cellular tumor grading systems and their modifications, as there seems to be room left for improvement concerning validity and applicability. Combining all results of the respective site by summation into one malignancy score revealed only the tumor surface malignancy score to be a significant predictor of OS and RFS. However, all considerations formerly mentioned taken into account, we conclude that its predictive power is mainly derived from single items being of high diagnostic value and at least single components of this score need further modifications.

RM as a metric or grouping parameter could not be identified as a valid prognostic factor for any of the outcome parameters investigated. Consecutively, we advocate the necessity of obtaining initial free RM with sufficient safety margins, but in the same moment would like to emphasize the importance of evaluating additional risk factors in each patient in order to select best treatment options.

DOI on the other hand was found to be a significant predictor for OS, RFS, and LM in metric and grouped analysis, and its integrations into standard staging procedure seem to have improved diagnostic quality.

## Clinical relevance

In conclusion, DOI evolved as the single most important parameter that can be obtained during histopathological examination of tissue slides. Consecutively, staging indeed seems to be more important than grading. However, applicability and prognostic relevance of grading parameters can be improved, e.g., by making them more reproducible using binary scales. Care must be taken to establish standardized procedures from the moment of taking the biopsy until processing the tissue and clear definitions of DOI must be followed in order to obtain valid results.

## Abbreviations

2-year survival rate: 2YSR
5-year survival rate: 5YSR
DOI: depth of invasion
ECS: extracapsular spread
HE: hematoxylin and eosin
HNSCC: head and neck squamous cell carcinoma
HPF: high power field
ICG: invasive cell grading
IF: invasion front
LM: lymphonodular metastasis
OS: overall survival
OSCC: oral squamous cell carcinoma
PD-L1: programmed death ligand 1
POG: pattern of growth
POI: pattern of invasion
RM: resection margin
RFS: relapse-free survival
TS: tumor surface
